# Supplementary material for: Associations between big five personality traits, facets, and sexual fantasies
Source: PLoS One. 2026 Feb 4;21(2):e0329745. doi: 10.1371/journal.pone.0329745 (PMC12871982; doi:10.1371/journal.pone.0329745)
Supplement: Attachment — Submitted filename: PONE-D-25-12929_R1_reviewer_tcsw27.pdf [file pone.0329745.s004.pdf]

PLOS ONE

Associations between Big Five Personality Traits, Facets, and Sexual Fantasies

--Manuscript Draft--

**Manuscript Number:**	PONE-D-25-12929R1
**Article Type:**	Research Article
**Full Title:**	Associations between Big Five Personality Traits, Facets, and Sexual Fantasies
**Short Title:**	PERSONALITY AND SEXUAL FANTASIES
**Corresponding Author:**	William J. Chopik<br>Michigan State University<br>East Lansing, MI UNITED STATES OF AMERICA
**Keywords:**	Big Five personality traits; personality facets; sexual fantasy; sexuality; sexual health and well-being
**Abstract:**	The present study investigated associations between Big Five personality traits, their facets, and the frequency and variety of sexual fantasies in a sample of 5,225 adults (M = 58.30 years old; 56.5% men). People high in conscientiousness and agreeableness report fewer sexual fantasies across exploratory, intimate, impersonal, and sadomasochistic domains; these effects were primarily driven by respectfulness and responsibility facets. Negative emotionality, particularly the depression facet, was associated with more frequent sexual fantasizing. Extraversion and open-mindedness showed minimal relationships with sexual fantasy frequency. These results underscore the importance of personality dimensions in understanding sexual thoughts, emphasizing the heterogeneity of sexual fantasies across individuals. Having a broader appreciation of the heterogeneity of sexual experiences can foster more inclusive approaches to sexual well-being in psychological research and clinical practice.
**Order of Authors:**	Emily Cannoot
	Amy C Moors
	William J. Chopik
**Response to Reviewers:**	Response to Reviewers (see appended letter for correct formatting)<br>We would like to thank the editor and reviewers for their thoughtful comments on the manuscript. We very much appreciate the constructive feedback and believe that the manuscript has improved significantly as a result of their suggestions. Below, we report how each of the reviewer issues was addressed and the corresponding changes to the manuscript. The reviewer comments are non-bolded, and our responses are bolded. We are happy to make any additional changes recommended by the Editor.<br><br>Editor<br><br>One reviewer supports the manuscript as is, while the other suggests several minor but important revisions that need to be addressed. These include clarifying the theoretical framework, elaborating on demographic variables, and improving the reporting of limitations. Please revise the manuscript accordingly.<br><br>Thank you for handling our manuscript. In evaluating the reviewers' comments, we elected to add details related to the inclusion/exclusion criteria in the Method (p 7) and to add a brief remark on the Implications of the study (see pg 12-13).<br><br>Reviewer #1<br>The paper addresses an important topic related to stress and resilience. However, some areas need improvement. Below is some feedback for improvement.<br>- Add literature review for second objective association between nurse's demographic characteristics and both stress and resilience (eg: gender, age)<br>- State what is the theory being used in this study.<br>- Methodology- What is the inclusion and exclusion criteria for the targeted sample.<br>- Add more demographic characteristics in the table related to the study (eg: age)<br>- Limitation, hanging sentence. "As such"

Powered by Editorial Manager® and ProduXion Manager® from Aries Systems Corporation

	- Add implication and conclusion related to the second objective of the study<br><br>We thank the reviewer for taking the time to provide a review. In discussions with the Editor, they partially left it up to our discretion for how to integrate comments from this review. After reviewing the comments, we ultimately decided to add information related to our inclusion/exclusion criteria (see p 7) and about the implications of our study (see pages 12-13). The other remarks fell a bit beyond the purview of our study or did not correspond to our manuscript, so we elected not to make those particular changes.<br><br>Reviewer #2<br>This interesting study of 5,225 adults examined the links between Big Five personality traits and sexual fantasies. High conscientiousness and agreeableness, driven by the facets of respectfulness and responsibility, were associated with fewer fantasies across various domains. Neuroticism, particularly the depression facet, correlated with more frequent fantasizing, while extraversion and open-mindedness showed minimal impact. The well-conducted study's findings reveal how personality shapes sexual thoughts, aiding clinicians in fostering sex-positive therapy. It highlights the diversity of sexual fantasies and supports inclusive approaches to sexual well-being.<br><br>We thank the reviewer for their positive evaluation of our manuscript!
**Additional Information:**	
**Question**	**Response**
**Financial Disclosure**<br><br>Enter a financial disclosure statement that describes the sources of funding for the work included in this submission. Review the submission guidelines for detailed requirements. View published research articles from *PLOS ONE* for specific examples.<br><br>This statement is required for submission and **will appear in the published article** if the submission is accepted. Please make sure it is accurate.<br><br>**Funded studies**<br>Enter a statement with the following details:<br>• Initials of the authors who received each award<br>• Grant numbers awarded to each author<br>• The full name of each funder<br>• URL of each funder website<br>• Did the sponsors or funders play any role in the study design, data collection and analysis, decision to publish, or preparation of the manuscript?<br><br>Did you receive funding for this work?	The author(s) received no specific funding for this work.
**Competing Interests**	The authors have declared that no competing interests exist.

Powered by Editorial Manager® and ProduXion Manager® from Aries Systems Corporation

Use the instructions below to enter a competing interest statement for this submission. On behalf of all authors, disclose any competing interests that could be perceived to bias this work—acknowledging all financial support and any other relevant financial or non-financial competing interests.<br><br>This statement is required for submission and **will appear in the published article** if the submission is accepted. Please make sure it is accurate and that any funding sources listed in your Funding Information later in the submission form are also declared in your Financial Disclosure statement.<br><br>View published research articles from *PLOS ONE* for specific examples.<br><br>**NO authors have competing interests**<br><br>Enter: *The authors have declared that no competing interests exist.*<br><br>**Authors with competing interests**<br><br>Enter competing interest details beginning with this statement:<br><br>*I have read the journal's policy and the authors of this manuscript have the following competing interests: [insert competing interests here]*<br><br>* typeset	
**Ethics Statement**<br><br>Enter an ethics statement for this submission. This statement is required if the study involved:<br><br>• Human participants<br>• Human specimens or tissue<br>• Vertebrate animals or cephalopods<br>• Vertebrate embryos or tissues	This study was carried out in accordance with the recommendations of Michigan State University's Institutional Review Board (IRB# x16-1291e) and run online with informed consent being secured from all participants (by clicking a next arrow; documentation requirement waived). Data were analyzed anonymously.

• Field research<br><br>Write "N/A" if the submission does not require an ethics statement.<br><br>General guidance is provided below. Consult the submission guidelines for detailed instructions. **Make sure that all information entered here is included in the Methods section of the manuscript.**	

Powered by Editorial Manager® and ProduXion Manager® from Aries Systems Corporation

**Format for specific study types**<br><br>**Human Subject Research (involving human participants and/or tissue)**<br>• Give the name of the institutional review board or ethics committee that approved the study<br>• Include the approval number and/or a statement indicating approval of this research<br>• Indicate the form of consent obtained (written/oral) or the reason that consent was not obtained (e.g. the data were analyzed anonymously)<br><br>**Animal Research (involving vertebrate animals, embryos or tissues)**<br>• Provide the name of the Institutional Animal Care and Use Committee (IACUC) or other relevant ethics board that reviewed the study protocol, and indicate whether they approved this research or granted a formal waiver of ethical approval<br>• Include an approval number if one was obtained<br>• If the study involved *non-human primates*, add *additional details* about animal welfare and steps taken to ameliorate suffering<br>• If anesthesia, euthanasia, or any kind of animal sacrifice is part of the study, include briefly which substances and/or methods were applied<br><br>**Field Research**<br><br>Include the following details if this study involves the collection of plant, animal, or other materials from a natural setting:<br>• Field permit number<br>• Name of the institution or relevant body that granted permission	
**Data Availability**<br><br>Authors are required to make all data underlying the findings described fully available, without restriction, and from the time of publication. PLOS allows rare exceptions to address legal and ethical concerns. See the PLOS Data Policy and FAQ for detailed information.	Yes - all data are fully available without restriction

A Data Availability Statement describing where the data can be found is required at submission. Your answers to this question constitute the Data Availability Statement and **will be published in the article**, if accepted.<br><br>**Important:** Stating 'data available on request from the author' is not sufficient. If your data are only available upon request, select 'No' for the first question and explain your exceptional situation in the text box.<br><br>Do the authors confirm that all data underlying the findings described in their manuscript are fully available without restriction?	
**Describe where the data may be found in full sentences. If you are copying our sample text, replace any instances of XXX with the appropriate details.**<br><br>• If the data are **held or will be held in a public repository**, include URLs, accession numbers or DOIs. If this information will only be available after acceptance, indicate this by ticking the box below. For example: *All XXX files are available from the XXX database (accession number(s) XXX, XXX.*).<br>• If the data are all contained **within the manuscript and/or Supporting Information files**, enter the following: *All relevant data are within the manuscript and its Supporting Information files.*<br>• If neither of these applies but you are able to provide **details of access elsewhere**, with or without limitations, please do so. For example:<br><br>*Data cannot be shared publicly because of [XXX]. Data are available from the XXX Institutional Data Access / Ethics Committee (contact via XXX) for researchers who meet the criteria for access to confidential data.*<br><br>*The data underlying the results presented in the study are available from (include the name of the third party*	All data, syntax, and materials can be found at https://osf.io/c9pzj/?view_only=b9672f35521f483c90a84701dfb105fc

and contact information or URL).<br>• This text is appropriate if the data are owned by a third party and authors do not have permission to share the data.<br><br>* typeset	
Additional data availability information:	

Manuscript Click here to access/download;Manuscript;Manuscript.docx

Associations between Big Five Personality Traits, Facets, and Sexual Fantasies

Emily Cannoot[1], Amy C. Moors[2,3], William J. Chopik[1*]

[1]Department of Psychology, Michigan State University, East Lansing, Michigan, United States of America

[2]Department of Psychology, Chapman University, Orange, California, United States of America

[3]The Kinsey Institute, Indiana University, Bloomington, Indiana, United States of America

* Corresponding author

Email: chopikwi@msu.edu (WJC)

Abstract

The present study investigated associations between Big Five personality traits, their facets, and the frequency and variety of sexual fantasies in a sample of 5,225 adults (*M* = 58.30 years old; 56.5% men). People high in conscientiousness and agreeableness report fewer sexual fantasies across exploratory, intimate, impersonal, and sadomasochistic domains; these effects were primarily driven by respectfulness and responsibility facets. Negative emotionality, particularly the depression facet, was associated with more frequent sexual fantasizing. Extraversion and open-mindedness showed minimal relationships with sexual fantasy frequency. These results underscore the importance of personality dimensions in understanding sexual thoughts, emphasizing the heterogeneity of sexual fantasies across individuals. Having a broader appreciation of the heterogeneity of sexual experiences can foster more inclusive approaches to sexual well-being in psychological research and clinical practice.

Keywords: Big Five personality traits, personality facets, sexual fantasy, sexuality, sexual health and well-being

Introduction

Sexual fantasies are common yet rarely discussed phenomena, often because of stigma and embarrassment around experiencing them [1, 2]. Because they are less often discussed, there is some ignorance about how common sexual fantasies are, who is most likely to have them, and if types of fantasies vary according to people's psychological traits. Among the little work done examining these questions, most has focused on broad indicators of personality while overlooking facet-level information on personality traits. Examining specific traits might provide a more complete description of "who" has sexual fantasies. The current study examined associations between Big Five personality traits and facets and the frequency and variety of sexual fantasies in a sample of over 5,000 adults.

Sexual fantasies and their role in people's lives

In the broadest sense, sexual fantasies are defined as "any mental imagery that is sexually arousing or erotic to the individual" [1]. Common taxonomies characterize sexual fantasies as multidimensional and generally varying several domains including romantic, experimental, personal/impersonal, masochistic, and force dominance-related fantasies [3]. Although there is some uncertainty about the exact origin of sexual fantasies, there is at least some acknowledgement that they are at least partially shaped by social and societal processes [1, 2, 4]. Most people admit to fantasizing about sex and enjoy doing so, regardless of their age, gender identity, or sexual orientation [1, 2, 5]. Nevertheless, there are a few demographic differences. Specifically, men tend to fantasize more than women, but there is some evidence that women may be more likely to engage in fulfilling these fantasies, particularly more romantic/intimate fantasies, although actual engagement is less commonly studied [3, 6]. Sexual fantasies are also more common among younger adults [7].

When trying to assess how sexual fantasies are associated with psychological characteristics, part of the difficulty in doing so is a historical framing of sexual fantasies as problematic or as reflective of psychopathology. In fact, many studies have focused on associations between sexual fantasies and exclusively antisocial behavior like aggressive tendencies or sex offending [8, 9]. However, the experience of sexual fantasies is so heterogeneous that merely knowing a person's sexual fantasies does not necessarily map on to their mental health characteristics [9, 10]. In other words, non-aggressive people could have aggressive sexual fantasies; interpersonally dominant people might have submissive sexual fantasies; introverted people might have voyeuristic fantasies.

Given that sex is one of the more underappreciated sources of well-being in people's lives [11], there is evidence to suggest that more frequent sexual fantasizing is associated with relationship promoting behaviors (e.g., verbalizing affection) and positive relationship outcomes [12-14]. Health and well-being benefits accompany positive and fulfilling relationships, so researchers have turned toward more deliberately included considerations of sex and sexuality in the study of health and well-being across the lifespan [7, 15]. Given that sexual fantasies are both common and might have some positive effects on people's happiness and relationships, what personality characteristics predict endorsement of various sexual fantasies?

Personality traits and sexual fantasy

Personality traits reflect people's characteristic patterns of thoughts, feelings, and behaviors. Currently, there is a general consensus regarding the Big Five as a framework for understanding variation in people's personalities [16]. The Big Five personality traits are comprised of extraversion, agreeableness, conscientiousness, negative emotionality (i.e., neuroticism), and open-mindedness (i.e., openness to experience)[17]. Among the many life

outcomes linked with personality, neuroticism is associated with more sexual dissatisfaction, negative emotions about sex, and symptoms of sexual dysfunction; openness is associated with more liberal attitudes toward sex [18]. Although the Big Five framework is the most commonly studied set of higher-order traits, there have been relatively few studies examining their associations with sexual fantasies (and no studies examining personality *facets* and sexual fantasies, from what we could find). In fact, there is little information *at all* about how sexual fantasizing is associated with any psychological traits (especially non-psychopathological traits).

This lack of research is apparent in one of the largest meta-analyses on personality and sexual behavior to date [18]. Among the 137 studies included in that paper, the authors could only identify between two to five effect sizes that implicated sexual fantasies, depending on the trait being studied. They found that people higher in openness (r = 24), extraversion (r = .10), and neuroticism (r = .13) tended to fantasize more often, although each of these studies collapsed across different types of sexual fantasies. Other studies have looked at more isolated traits (e.g., sensation seeking) that could reasonably be subsumed under the Big Five [19-21]. In the work conducted since that 2018 meta-analysis, a more complicated picture of the association between personality and sexual fantasies has emerged, sometimes contradicting the meta-analysis. For example, extraverts and open-minded individuals are more likely to fantasize about extra-dyadic sex and consensual non-monogamy; people high in neuroticism were less likely to report these types of fantasies [10, 22]. Yet, other research suggests that introverts might have more sexual fantasies than extraverts [23]. People high in conscientiousness or agreeableness were less likely to have aggressive sexual fantasies than those low in these traits [8, 24]. Highly neurotic people tend to have both more positive *and* negative sexual thoughts, including violent fantasies [4, 23, 25]. People open to experience (i.e., open mindedness) tend to sexually daydream more often

than those less open to experience [26]. Yet, other studies find few or no associations between personality and sexual fantasies of various types [27]. We revisited this question in a large sample of adults who reported on their personalities and how much they engaged in a variety of sexual fantasies.

One additional contribution our study makes to the literature is examining associations between sexual fantasies and personality *facets*. Personality facets are lower order traits, subsumed by the Big Five, that provide more specific information about people's personalities [19, 28]. Integrating facets into an analysis can reveal more complete insights into who is most likely to have certain sexual fantasies. For example, if extraverts have more sexual fantasies [22], is it primarily driven by their sociability, their assertiveness, or the energy level (all facets of extraversion)? Likewise, if conscientious people are less likely to have aggressive fantasies [8], it is likely because of the respectfulness and compassionate facets of the trait rather than the trust facet per se. In addition to focusing on links between people's personality facets and sexual fantasies, we ran supplementary analyses in which we controlled for the covariation among the Big Five traits in their associations with sexual fantasies. Given the mixed results from previous research, and the lack of research about facets, we treated all analyses as exploratory.

Method

The present exploratory study was not preregistered. All data, syntax, and materials can be found at https://osf.io/c9pzj/?view_only=b9672f35521f483c90a84701dfb105fc This study was carried out in accordance with the recommendations of Michigan State University's Institutional Review Board (IRB# x16-1291e) and run online with informed consent being secured from all participants (by clicking a next arrow; documentation requirement waived). Data were analyzed anonymously.

Participants and procedure

Participants comprised of 5,225 internet respondents recruited from Qualtrics Panels who took part in an online study about close relationships in exchange for $10. Data were collected from March 15, 2021 through October 21, 2021. They ranged in age from 18 to 94 (M_{age} = 58.30, *SD* = 15.93). The sample was 56.5% men, 43.2% women, and .3% who described themselves as another gender. The sample was 87.7% White, 3.5% Asian, 3.2% Hispanic/Latinx, 3.1% Black/African American, and 2.5% other races/ethnicities.

Given the scope of the present study focused on close relationships, all participants were either married (94.5%) or dating (5.5%). Mean relationship length was 29.68 years (*SD* = 16.93). The sample was relatively sexually active, with 68.6% of participants having sex at least once per month. The inclusion criteria were that people were currently in a relationship and at least 18 years old. We collected as many participants as funding allowed (see below for power analysis though). We did not exclude any participants, and missing data were handled via listwise deletion.

Measures

Big Five personality traits

The Big Five Personality traits were measured using the short-form version of the Big Five-Inventory-2 [29]. The questionnaire contains 30 items (6 for each trait), and individuals respond to each statement according to how well it describes them on a scale ranging from 1 (*disagree strongly*) to 5 (*agree strongly*). Mean composites were computed for extraversion (e.g., “I am someone who is outgoing, sociable;” α = .71, *M* = 3.27, *SD* = .72), agreeableness (e.g., “I

am someone who is compassionate, has a soft heart;" *α* = .81, *M* = 3.85, *SD* = .71), conscientiousness (e.g., "I am someone who is persistent, works until the task is finished;" *α* = .80, *M* = 3.93, *SD* = .73), negative emotionality (e.g., "I am someone who worries a lot;" *α* = .84, *M* = 2.37, *SD* = .84), and open-mindedness (e.g., "I am someone who is fascinated by art, music, or literature;" *α* = .73, *M* = 3.37, *SD* = .73).

Within each of the broader Big Five personality traits are three facets—smaller, more specific descriptors of personality. We also calculated the means for three facets (measured with two items each) of extraversion (sociability (*α* = .63), assertiveness (*α* = .65), and energy level (*α* = .68)), agreeableness (compassion (*α* = .61), respectfulness (*α* = .57), and trust (*α* = .61)), conscientiousness (organization (*α* = .74), productiveness (*α* = .59), and responsibility (*α* = .46)), negative emotionality (anxiety (*α* = .67), depression (*α* = .67), and emotional volatility (*α* = .63)), and open-mindedness (aesthetic sensitivity (*α* = .53), intellectual curiosity (*α* = .45), and creative imagination (*α* = .56)).

Sexual fantasies

Sexual fantasies were assessed with the Sexual Fantasies Questionnaire [30], which asked participants to indicate how often they fantasized about each of 40 themes on a scale ranging from 1 (*never*) to 6 (*daily*). In addition to an overall composite of sexual fantasizing (*α* = .97, *M* = 1.73, *SD* = .90), the responses can be subsumed into four 10-item composite scales: exploratory sexual fantasies (e.g., "participating in an orgy;" *α* = .94, *M* = 1.52, *SD* = .94), intimate sexual fantasies (e.g., "making love outdoors in a romantic setting;" *α* = .93, *M* = 2.45, *SD* = 1.21), impersonal sexual fantasies (e.g., "watching others have sex;" *α* = .91, *M* = 1.58, *SD* = .90), and sadomasochistic sexual fantasies (e.g., "being forced to do something;" *α* = .95, *M* = 1.37, *SD* = .89).

Analytical Approach

We began by running bivariate associations between personality traits, facets, and the different sexual fantasy outcomes (i.e., a composite total and each of the four composite scales). Then, to account for the covariation, we ran follow-up linear regressions that included all personality traits, age, and gender. From these models, partial correlations were extracted. For these models that featured control variables, we ran two models, one for the Big Five traits and another for the (15) facets. These were done separately because the broader Big Five are made up of the facets and including them all in one model would introduce multicollinearity problems. This information is presented in Table 1 in the form of partial correlations.

Results

With our sample size of 5,225 people, we could detect effects as small as $r = .039$ with 80% power at $\alpha = .05$. Because of the large sample size, many small correlations (even those slightly smaller than $r = .039$) were statistically significant at $p < .05$ (all $rs > |.03|$ and $rs_p > |.027|$). Thus, we primarily focus on the magnitude of the correlations and highlight the largest and most robust associations.

As seen in Table 1, the most consistent predictor of sexual fantasies was conscientiousness. Higher levels of conscientiousness were associated with a lower frequency of all four types of sexual fantasies (exploration, intimate, impersonal, and sadomasochistic). The next most consistent predictor was agreeableness; higher levels of agreeableness associated with a lower frequency of most types of fantasies. Extraverts tended to fantasize about all four types of sexual fantasies more, but only *after* the covariation between traits was controlled for. People with higher levels of negative emotionality tended to fantasize about all four types of sexual fantasies. Open-mindedness was mostly unrelated to fantasies, particularly after controlling for

covariation with other traits, age, and gender. One consistent pattern that emerged is that associations between personality traits and sexual fantasy were reduced after controlling for covariation between the traits and demographics (i.e., comparing the bivariate and partial correlation columns in Table 1).

Table 1. Bivariate and Partial Correlations between Personality and Sexual Fantasies

Construct	Fantasy Total		Exploration		Intimate		Impersonal		Sadomasochistic	
	Bivariate	Partial	Bivariate	Partial	Bivariate	Partial	Bivariate	Partial	Bivariate	Partial
Extraversion	-0.001	0.058	-0.033	0.037	0.081	0.085	-0.034	0.037	-0.041	0.041
Agreeableness	-0.228	-0.083	-0.243	-0.098	-0.123	-0.015	-0.260	-0.110	-0.233	-0.089
Conscientiousness	-0.265	-0.131	-0.278	-0.141	-0.158	-0.068	-0.270	-0.119	-0.286	-0.150
Negative Emotionality	0.171	0.038	0.173	0.023	0.100	0.051	0.180	0.042	0.189	0.016
Open Mindedness	-0.005	0.023	-0.033	0.008	0.080	0.075	-0.042	0.004	-0.052	-0.026
Sociability	0.004	0.061	-0.007	0.057	0.040	0.051	-0.016	0.053	-0.008	0.058
Assertiveness	0.004	-0.031	-0.020	-0.035	0.073	0.001	-0.011	-0.033	-0.045	-0.047
Energy Level	-0.011	0.086	-0.046	0.064	0.067	0.087	-0.047	0.064	-0.039	0.081
Compassion	-0.201	-0.021	-0.212	-0.024	-0.111	-0.002	-0.226	-0.029	-0.207	-0.026
Respectfulness	-0.258	-0.085	-0.271	-0.093	-0.149	-0.034	-0.276	-0.085	-0.272	-0.103
Trust	-0.115	0.028	-0.126	0.024	-0.052	0.026	-0.148	0.006	-0.108	0.045
Organization	-0.187	0.005	-0.194	0.005	-0.126	-0.008	-0.183	0.016	-0.193	0.010
Productiveness	-0.205	-0.034	-0.221	-0.037	-0.106	-0.017	-0.216	-0.035	-0.230	-0.038
Responsibility	-0.283	-0.055	-0.294	-0.061	-0.168	-0.016	-0.289	-0.054	-0.306	-0.071
Anxiety	0.070	-0.081	0.075	-0.087	0.032	-0.025	0.078	-0.085	0.081	-0.101
Depression	0.384	0.276	0.380	0.270	0.263	0.171	0.382	0.274	0.402	0.290
Emotional Volatility	0.185	0.003	0.182	-0.005	0.123	0.013	0.190	0.007	0.196	-0.007
Aesthetic Sensitivity	0.039	0.055	0.020	0.054	0.086	0.055	0.004	0.043	0.012	0.037
Intellectual Curiosity	-0.005	-0.020	-0.025	-0.021	0.064	0.005	-0.030	-0.025	-0.050	-0.041
Creative Imagination	-0.051	-0.010	-0.078	-0.026	0.037	0.022	-0.078	-0.016	-0.092	-0.027

Note. Correlations r > |.03| and r_p > |.027| are significant at p < .05. More intensely green cells are more strongly positive correlations. More intensely red cells are more strongly negative correlations.

In the bottom portion of Table 1 (run in separate models than the higher-order traits), the facet most largely correlated with higher levels of frequent fantasizing in all categories was depression (a facet of negative emotionality), such that people with more depressive personalities tended to fantasize about all four types of sexual fantasies. Respectfulness (for agreeableness) and responsibility (for conscientiousness) had the strongest negative associations with each type of sexual fantasy, although the magnitude of these associations was reduced dramatically in the covariate analysis. Most of the remaining facet associations had correlations smaller than $r = |.20|$, and many associations either became non-significant or even switched signs (albeit these associations were near-zero) after controlling for demographic characteristics and the covariation with the other facets. One surprising finding is that the open-mindedness facets, including creative imagination, were largely unrelated to any type of sexual fantasy, which one might expect because open people tend to fantasize about both sexual and non-sexual things more often [26, 31].

Discussion

We examined associations between sexual fantasies and the Big Five personality traits and facets. People high in agreeableness and conscientiousness reported less frequent sexual fantasies, both overall and for specific types of fantasies (e.g., exploration, intimate, impersonal, sadomasochistic). People high in negative emotionality tended to fantasize more. Measuring facets proved worthwhile in that we were able to discern the parts or facets of these traits that are related to sexual fantasy. Specifically, those with depressive personalities reported more frequent sexual fantasies. Those who were high in respectfulness and responsibility reported less frequent sexual fantasies. One implication of the current work is that individual differences in personality might be useful in predicting variation in sexual fantasy frequencies, although they are not

wholly redundant with each other (and some associations are relatively small or modest). Knowing these associations further advances the predictive power of personality while showing that variation in sexual fantasies are common.

Big Five personality and sexual fantasies

Despite most people experiencing sexual fantasies, sexual fantasies are relatively understudied or framed as pathological [1, 9]. Research shows the utility in approaching the study of sexual fantasies in a value-neutral way has revealed that sexual fantasies are often linked to positive relationship and life outcomes and are also common across the lifespan [7, 12-15]. There are relatively few studies examining associations between psychological predictors, like the Big Five personality traits [20]. However, those studies largely showed that people higher in agreeableness and conscientiousness are less likely to report sexual fantasizing, people higher in openness might be more likely to have sexual fantasies in some contexts, and people high in negative emotionality were more likely to have a variety of sexual fantasies [10, 20, 22, 25, 26].

We were able to replicate some of these findings but not others. Indeed, people higher in agreeableness or conscientiousness tended to report less frequent sexual fantasies. Although negative emotionality was associated with more frequent sexual fantasies (and sexual fantasies of different types), these associations dropped to near-zero (and in many cases non-significance). Openness to experience was largely unrelated to sexual fantasies (except a positive association with intimate sexual fantasies). Extraversion had a small positive association with sexual fantasies, but this mostly only occurred in the models with covariates (and the signs of the associations changed directions). Thus, the main take-aways for the broader trait level are that

agreeableness and conscientiousness are negatively related to sexual fantasy frequencies; extraversion and negative emotionality are positively related to sexual fantasy frequencies—although it mostly depends on whether covariates are controlled for; and openness is mostly unrelated to sexual fantasies. These findings were seen across different types of fantasies, whether they were exploratory, intimate, impersonal, or sadomasochistic in nature. Agreeableness and conscientiousness are associated with norm endorsement, harm prevention, and traditionalism [32, 33]. Thus, it seems reasonable to assume that they might be less likely to engage in fantasies that are non-traditional, bridge social norms, or simulate consensual aggression.

The findings with respect to negative emotionality were a bit harder to interpret. People high in negative emotionality are more likely to focus on negative stimuli, perseverate, and have negative thoughts overall [34, 35]. They are also more likely to experience various types of disgust, including sexual disgust [25, 36]. These observations together might be why they are more likely to think about certain types of sexual fantasies (e.g., sadomasochistic), but this does not explain why they are prone to more ostensibly positive or neutral fantasies as well. However, our study merely examined the frequency of sexual fantasies and not their utility. In other words, some researchers have suggested that people high in negative emotionality might engage in sexual fantasies as an emotion regulation tool to compensate for negative mood [24]. Indeed, when sexual fantasies are operationalized in terms of valence (e.g., positive or negative fantasies, people high in negative emotionality tend to have both positive and negative sexual thoughts and fantasies [24, 25]. Thus, people high in negative emotionality tend to fantasize more overall, such that they have both positive and negative sexual fantasies.

Facets and fantasy

Examining associations between Big Five personality facets (or subcomponents of the broader traits) enabled a more focused examination. Knowing more about these subcomponents has significantly advanced our understanding of personality-outcome associations [28, 37-39]. Although some of the Big Five traits were associated with sexual fantasies, it is possible that there are subcomponents of those larger traits that can give us a clue for why people fantasize more or less.

Indeed, respectfulness (for agreeableness) and responsibility (for conscientiousness) were the drivers of the negative associations seen among their higher-order traits. What this reveals is that agreeable people may be less likely to sexually fantasize because of their respect for norms and others (hence the less common sadomasochistic fantasies) and not that tender feelings about others or perceptions of trust are holding them back from doing so (particularly in the covariate analyses). Likewise, people high in responsibility are likely to moralize themselves and others and avoid sexual thinking (and maybe sex) altogether [18]. What the facet analysis also tells us is that people who are more or less organized (or more or less productive) are no more or less likely to sexually fantasize. The large association with depression (a facet of negative emotionality) can also reveal some reasons why negative emotionality was positively associated with sexual fantasies. Specifically, the fact that depression was strongly related to sexual fantasy—and that anxiety and emotional volatility were not—provides support for the possible emotional regulatory function of sexual fantasies [24, 25]. In other words, it is not the case that emotionally volatile people or anxious people were more likely to sexually fantasize (if anything, anxious people were slightly less likely to fantasize in some models). Rather, those more prone to negative emotionality were also those most likely to sexually fantasize, possibly as a way to have more positive cognitions [25]. The fact that open-mindedness was largely unrelated to sexual

fantasies was a bit surprising, particularly given past research [26, 31]. There is a precedent for null results in this literature [27], and future research can examine if these results might be attributable to the way open-mindedness is operationalized [40].

Limitations and future directions

This study had many strengths, including having a large sample of adults providing information on their personality facets and a variety of sexual fantasies. Nevertheless, some limitations must be discussed.

First, we relied entirely on self-report data, which may be prone to bias [41], particularly when discussing sensitive topics like sexual fantasies, which many people find embarrassing or uncomfortable to discuss [2]. Worth noting, associations between personality and sexual fantasies and thoughts tend to transcend differences in social desirability [24]. Also, the anonymity of our questionnaire likely also reduced these concerns. Nevertheless, future research can investigate ways to circumvent the social desirability effects that accompany self-reports about sexual fantasies. We also hope that this future work recruits a more diverse sample, including those from non-US countries and among people with different relationship statuses (e.g., single people, partnered people, non-monogamous people).

Second, our data were cross-sectional and was merely a snapshot of people's sexual fantasies and how they were related to personality traits. Stability information on sexual fantasies is relatively rare, but they do tend to fluctuate over the course of a few weeks and months [42, 43] and tend to differ across the lifespan [7]. Future research can follow participants longitudinally to determine if personality traits prospective development of sexual fantasies. Further, it is possible that personality traits and sexual fantasies might co-develop together over

time such that fantasies might become more or less common (or different types of fantasies might become more salient) as people's personalities or life circumstances change.

Conclusion

The current study examined associations between personality traits and sexual fantasies. We found that people high in conscientiousness and agreeableness fantasized less, and people high in neuroticism fantasized more. Among the personality facets, those with higher in depressive, and lower in respectfulness and responsibility facets, were more likely to sexually fantasize. Although sexual fantasies are common among the general population [1, 9], we found that people do not homogeneously report various types of sexual fantasy. Knowing which personality traits are associated with different types of sexual fantasies will likely provide clinicians and mental health professionals with additional information in providing sex-positive therapeutic settings that acknowledge people's sexual thoughts—an effort that is increasingly becoming more common [7, 14].

References

1. Leitenberg, H. and K. Henning, *Sexual fantasy.* Psychological bulletin, 1995. **117**(3): p. 469.
2. Ziegler, A. and T.D. Conley, *The importance and meaning of sexual fantasies in intimate relationships*, in *The psychology of love and hate in intimate relationships*, K. Aumer, Editor. 2016. p. 29-45.
3. Wilson, G.D. and R.J. Lang, *Sex differences in sexual fantasy patterns.* Personality and Individual Differences, 1981. **2**(4): p. 343-346.
4. Hawley, P.H. and W.A. Hensley IV, *Social dominance and forceful submission fantasies: Feminine pathology or power?* Journal of Sex Research, 2009. **46**(6): p. 568-585.
5. Leistner, C.E., et al., *The Co-existence of Love and Desire: Experiences of Bisexual, Lesbian, and Heterosexual Women.* Sexuality & Culture, 2022. **26**(2): p. 531-547.
6. Bogaert, A.F., B.A. Visser, and J.A. Pozzebon, *Gender differences in object of desire self-consciousness sexual fantasies.* Archives of Sexual Behavior, 2015. **44**: p. 2299-2310.
7. Kolodziejczak, K., et al., *Sexual activity, sexual thoughts, and intimacy among older adults: Links with physical health and psychosocial resources for successful aging.* Psychology and aging, 2019. **34**(3): p. 389.
8. Bondü, R. and J.B. Birke, *Aggression-related sexual fantasies: Prevalence rates, sex differences, and links with personality, attitudes, and behavior.* The journal of sexual medicine, 2021. **18**(8): p. 1383-1397.
9. Lehmiller, J.J. and A.M. Gormezano, *Sexual fantasy research: A contemporary review.* Current Opinion in Psychology, 2023. **49**: p. 101496.

10. Lehmiller, J.J., *Tell me what you want: The science of sexual desire and how it can help you improve your sex life.* 2018, Boston: Da Capo Lifelong Books.

11. Muise, A., U. Schimmack, and E.A. Impett, *Sexual frequency predicts greater well-being, but more is not always better.* Social Psychological and Personality Science, 2015. **7**(4): p. 295-302.

12. Busch, T.M., et al., *Exploring the Impact of Fantasizing on Romantic Relationships and Attachment.* Sexuality & Culture, 2024.

13. Birnbaum, G.E., et al., *What Fantasies Can Do to Your Relationship: The Effects of Sexual Fantasies on Couple Interactions.* Personality and Social Psychology Bulletin, 2019. **45**(3): p. 461-476.

14. Newbury, R., et al., *Sexual fantasy as a clinical intervention.* Sexual and Relationship Therapy, 2012. **27**(4): p. 358-371.

15. Kolodziejczak, K., et al., *Perceived importance and enjoyment of sexuality in late midlife: Cohort differences in the Longitudinal Aging Study Amsterdam (LASA).* Sexuality Research and Social Policy, 2021. **18**: p. 621-635.

16. John, O.P. and S. Srivastava, *The Big Five trait taxonomy: History, measurement, and theoretical perspectives*, in *Handbook of personality: Theory and research*, L.A. Pervin and O.P. John, Editors. 1999. p. 102-138.

17. Soto, C.J. and O.P. John, *The next Big Five Inventory (BFI-2): Developing and assessing a hierarchical model with 15 facets to enhance bandwidth, fidelity, and predictive power.* Journal of Personality and Social Psychology, 2017. **113**(1): p. 117-143.

18. Allen, M.S. and E.E. Walter, *Linking big five personality traits to sexuality and sexual health: A meta-analytic review.* Psychological Bulletin, 2018. **144**(10): p. 1081-1110.

19. Bainbridge, T.F., S.G. Ludeke, and L.D. Smillie, *Evaluating the Big Five as an organizing framework for commonly used psychological trait scales.* Journal of Personality and Social Psychology, 2022. **122**(4): p. 749-777.

20. Nowak, B., *Big Five Personality Traits: Sexual Fantasies*, in *Encyclopedia of Sexual Psychology and Behavior*, T.K. Shackelford, Editor. 2023, Springer. p. 1-3.

21. Bivona, J.M., J.W. Critelli, and M.J. Clark, *Women's Rape Fantasies: An Empirical Evaluation of the Major Explanations.* Archives of Sexual Behavior, 2012. **41**(5): p. 1107-1119.

22. Lehmiller, J.J., *Fantasies About Consensual Nonmonogamy Among Persons in Monogamous Romantic Relationships.* Archives of Sexual Behavior, 2020. **49**(8): p. 2799-2812.

23. Sierra, J., S. Álvarez-Castro, and E. Miró, *Relación entre rasgos de personalidad y fantasías sexuales.* Terapia Psicológica, 1995. **3**: p. 7-12.

24. Egan, V. and V. Campbell, *Sensational interests, sustaining fantasies and personality predict physical aggression.* Personality and Individual Differences, 2009. **47**(5): p. 464-469.

25. Moyano, N. and J.C. Sierra, *Relationships between personality traits and positive/negative sexual cognitions.* International Journal of Clinical and Health Psychology, 2013. **13**(3): p. 189-196.

26. Pérez-González, S., N. Moyano, and J.C. Sierra, *La capacidad de ensoñación sexual: su relación con la actitud hacia las fantasías sexuales y rasgos de personalidad.* Cuadernos de Medicina Psicosomática y Psiquiatría de Enlace, 2011. **99**: p. 9-20.

27. Williams, K.M., et al., *Inferring Sexually Deviant Behavior From Corresponding*

Fantasies:The Role of Personality and Pornography Consumption. Criminal Justice and Behavior, 2009. **36**(2): p. 198-222.

28. Schwaba, T., et al., *A facet atlas: Visualizing networks that describe the blends, cores, and peripheries of personality structure.* PLOS ONE, 2020. **15**(7): p. e0236893.
29. Soto, C.J. and O.P. John, *Short and extra-short forms of the Big Five Inventory–2: The BFI-2-S and BFI-2-XS.* Journal of Research in Personality, 2017. **68**: p. 69-81.
30. Wilson, G.D., *The sex fantasy questionnaire: An update.* Sexual and Relationship Therapy, 2010. **25**(1): p. 68-72.
31. Sánchez-Bernardos, M.L., et al., *Fantasy Proneness and Personality Profiles.* Imagination, Cognition and Personality, 2015. **34**(4): p. 327-339.
32. Wilmot, M.P. and D.S. Ones, *Agreeableness and its consequences: A quantitative review of meta-analytic findings.* Personality and Social Psychology Review, 2022. **26**(3): p. 242-280.
33. Roberts, B.W., et al., *The structure of conscientiousness: An empirical investigation based on seven major personality questionnaires.* Personnel Psychology, 2005. **58**(1): p. 103-139.
34. Robinson, M.D., et al., *Stuck in a rut: perseverative response tendencies and the neuroticism-distress relationship.* Journal of Experimental Psychology: General, 2006. **135**(1): p. 78.
35. Clark, L.A. and D. Watson, *Temperament: An ongoing paradigm for trait psychology*, in *Handbook of Personality: Theory and Research*, O.P. John, R.W. Robins, and L.A. Pervin, Editors. 2008, The Guilford Press: New York. p. 265-286.
36. Olatunji, B.O., et al., *Core, animal reminder, and contamination disgust: Three kinds of*

disgust with distinct personality, behavioral, physiological, and clinical correlates. Journal of Research in Personality, 2008. **42**(5): p. 1243-1259.

37. Weidmann, R., et al., *Trait and facet personality similarity and relationship and life satisfaction in romantic couples.* Journal of Research in Personality, 2023. **104**: p. 104378.

38. Mund, M. and F.J. Neyer, *Treating personality-relationship transactions with respect: Narrow facets, advanced models, and extended time frames.* Journal of Personality and Social Psychology, 2014. **107**(2): p. 352-368.

39. Stewart, R.D., et al., *The finer details? The predictability of life outcomes from Big Five domains, facets, and nuances.* Journal of Personality, 2022. **90**(2): p. 167-182.

40. DeYoung, C.G., R.G. Grazioplene, and J.B. Peterson, *From madness to genius: The Openness/Intellect trait domain as a paradoxical simplex.* Journal of Research in Personality, 2012. **46**(1): p. 63-78.

41. Paulhus, D.L. and S. Vazire, *The self-report method*, in *Handbook of Research Methods in Personality Psychology*, R.W. Robins, R.C. Fraley, and R.F. Krueger, Editors. 2007, The Guilford Press: New York. p. 224-239.

42. Cascalheira, C.J., et al., *Changes in Sexual Fantasy and Solitary Sexual Practice During Social Lockdown Among Young Adults in the UK.* Sexual Medicine, 2021. **9**(3): p. 100342-100342.

43. Dawson, S.J., K.D. Suschinsky, and M.L. Lalumière, *Sexual Fantasies and Viewing Times Across the Menstrual Cycle: A Diary Study.* Archives of Sexual Behavior, 2012. **41**(1): p. 173-183.

Revised Manuscript with Track Changes

Associations between Big Five Personality Traits, Facets, and Sexual Fantasies

Emily Cannoot[1], Amy C. Moors[2,3], William J. Chopik[1*]

[1]Department of Psychology, Michigan State University, East Lansing, Michigan, United States of America

[2]Department of Psychology, Chapman University, Orange, California, United States of America

[3]The Kinsey Institute, Indiana University, Bloomington, Indiana, United States of America

* Corresponding author

Email: chopikwi@msu.edu (WJC)

Abstract

The present study investigated associations between Big Five personality traits, their facets, and the frequency and variety of sexual fantasies in a sample of 5,225 adults (M = 58.30 years old; 56.5% men). People high in conscientiousness and agreeableness report fewer sexual fantasies across exploratory, intimate, impersonal, and sadomasochistic domains; these effects were primarily driven by respectfulness and responsibility facets. Negative emotionality, particularly the depression facet, was associated with more frequent sexual fantasizing. Extraversion and open-mindedness showed minimal relationships with sexual fantasy frequency. These results underscore the importance of personality dimensions in understanding sexual thoughts, emphasizing the heterogeneity of sexual fantasies across individuals. Having a broader appreciation of the heterogeneity of sexual experiences can foster more inclusive approaches to sexual well-being in psychological research and clinical practice.

Keywords: Big Five personality traits, personality facets, sexual fantasy, sexuality, sexual health and well-being

Introduction

Sexual fantasies are common yet rarely discussed phenomena, often because of stigma and embarrassment around experiencing them [1, 2]. Because they are less often discussed, there is some ignorance about how common sexual fantasies are, who is most likely to have them, and if types of fantasies vary according to people's psychological traits. Among the little work done examining these questions, most has focused on broad indicators of personality while overlooking facet-level information on personality traits. Examining specific traits might provide a more complete description of "who" has sexual fantasies. The current study examined associations between Big Five personality traits and facets and the frequency and variety of sexual fantasies in a sample of over 5,000 adults.

Sexual fantasies and their role in people's lives

In the broadest sense, sexual fantasies are defined as "any mental imagery that is sexually arousing or erotic to the individual" [1]. Common taxonomies characterize sexual fantasies as multidimensional and generally varying several domains including romantic, experimental, personal/impersonal, masochistic, and force dominance-related fantasies [3]. Although there is some uncertainty about the exact origin of sexual fantasies, there is at least some acknowledgement that they are at least partially shaped by social and societal processes [1, 2, 4]. Most people admit to fantasizing about sex and enjoy doing so, regardless of their age, gender identity, or sexual orientation [1, 2, 5]. Nevertheless, there are a few demographic differences. Specifically, men tend to fantasize more than women, but there is some evidence that women may be more likely to engage in fulfilling these fantasies, particularly more romantic/intimate fantasies, although actual engagement is less commonly studied [3, 6]. Sexual fantasies are also more common among younger adults [7].

When trying to assess how sexual fantasies are associated with psychological characteristics, part of the difficulty in doing so is a historical framing of sexual fantasies as problematic or as reflective of psychopathology. In fact, many studies have focused on associations between sexual fantasies and exclusively antisocial behavior like aggressive tendencies or sex offending [8, 9]. However, the experience of sexual fantasies is so heterogeneous that merely knowing a person's sexual fantasies does not necessarily map on to their mental health characteristics [9, 10]. In other words, non-aggressive people could have aggressive sexual fantasies; interpersonally dominant people might have submissive sexual fantasies; introverted people might have voyeuristic fantasies.

Given that sex is one of the more underappreciated sources of well-being in people's lives [11], there is evidence to suggest that more frequent sexual fantasizing is associated with relationship promoting behaviors (e.g., verbalizing affection) and positive relationship outcomes [12-14]. Health and well-being benefits accompany positive and fulfilling relationships, so researchers have turned toward more deliberately included considerations of sex and sexuality in the study of health and well-being across the lifespan [7, 15]. Given that sexual fantasies are both common and might have some positive effects on people's happiness and relationships, what personality characteristics predict endorsement of various sexual fantasies?

Personality traits and sexual fantasy

Personality traits reflect people's characteristic patterns of thoughts, feelings, and behaviors. Currently, there is a general consensus regarding the Big Five as a framework for understanding variation in people's personalities [16]. The Big Five personality traits are comprised of extraversion, agreeableness, conscientiousness, negative emotionality (i.e., neuroticism), and open-mindedness (i.e., openness to experience)[17]. Among the many life

outcomes linked with personality, neuroticism is associated with more sexual dissatisfaction, negative emotions about sex, and symptoms of sexual dysfunction; openness is associated with more liberal attitudes toward sex [18]. Although the Big Five framework is the most commonly studied set of higher-order traits, there have been relatively few studies examining their associations with sexual fantasies (and no studies examining personality *facets* and sexual fantasies, from what we could find). In fact, there is little information *at all* about how sexual fantasizing is associated with any psychological traits (especially non-psychopathological traits).

This lack of research is apparent in one of the largest meta-analyses on personality and sexual behavior to date [18]. Among the 137 studies included in that paper, the authors could only identify between two to five effect sizes that implicated sexual fantasies, depending on the trait being studied. They found that people higher in openness (r = 24), extraversion (r = .10), and neuroticism (r = .13) tended to fantasize more often, although each of these studies collapsed across different types of sexual fantasies. Other studies have looked at more isolated traits (e.g., sensation seeking) that could reasonably be subsumed under the Big Five [19-21]. In the work conducted since that 2018 meta-analysis, a more complicated picture of the association between personality and sexual fantasies has emerged, sometimes contradicting the meta-analysis. For example, extraverts and open-minded individuals are more likely to fantasize about extra-dyadic sex and consensual non-monogamy; people high in neuroticism were less likely to report these types of fantasies [10, 22]. Yet, other research suggests that introverts might have more sexual fantasies than extraverts [23]. People high in conscientiousness or agreeableness were less likely to have aggressive sexual fantasies than those low in these traits [8, 24]. Highly neurotic people tend to have both more positive *and* negative sexual thoughts, including violent fantasies [4, 23, 25]. People open to experience (i.e., open mindedness) tend to sexually daydream more often

than those less open to experience [26]. Yet, other studies find few or no associations between personality and sexual fantasies of various types [27]. We revisited this question in a large sample of adults who reported on their personalities and how much they engaged in a variety of sexual fantasies.

One additional contribution our study makes to the literature is examining associations between sexual fantasies and personality *facets*. Personality facets are lower order traits, subsumed by the Big Five, that provide more specific information about people's personalities [19, 28]. Integrating facets into an analysis can reveal more complete insights into who is most likely to have certain sexual fantasies. For example, if extraverts have more sexual fantasies [22], is it primarily driven by their sociability, their assertiveness, or the energy level (all facets of extraversion)? Likewise, if conscientious people are less likely to have aggressive fantasies [8], it is likely because of the respectfulness and compassionate facets of the trait rather than the trust facet per se. In addition to focusing on links between people's personality facets and sexual fantasies, we ran supplementary analyses in which we controlled for the covariation among the Big Five traits in their associations with sexual fantasies. Given the mixed results from previous research, and the lack of research about facets, we treated all analyses as exploratory.

Method

The present exploratory study was not preregistered. All data, syntax, and materials can be found at https://osf.io/c9pzj/?view_only=b9672f35521f483c90a84701dfb105fc This study was carried out in accordance with the recommendations of Michigan State University's Institutional Review Board (IRB# x16-1291e) and run online with informed consent being secured from all participants (by clicking a next arrow; documentation requirement waived). Data were analyzed anonymously.

Formatted: Font: 12 pt

Formatted: Font: 12 pt

Participants and procedure

Participants comprised of 5,225 internet respondents recruited from Qualtrics Panels who took part in an online study about close relationships in exchange for $10. Data were collected from March 15, 2021 through October 21, 2021. They ranged in age from 18 to 94 (M_{age} = 58.30, *SD* = 15.93). The sample was 56.5% men, 43.2% women, and .3% who described themselves as another gender. The sample was 87.7% White, 3.5% Asian, 3.2% Hispanic/Latinx, 3.1% Black/African American, and 2.5% other races/ethnicities.

Given the scope of the present study focused on close relationships, all participants were either married (94.5%) or dating (5.5%). Mean relationship length was 29.68 years (*SD* = 16.93). The sample was relatively sexually active, with 68.6% of participants having sex at least once per month. The inclusion criteria were that people were currently in a relationship and at least 18 years old. We collected as many participants as funding allowed (see below for power analysis though). We did not exclude any participants, and missing data were handled via listwise deletion.

Measures

Big Five personality traits

The Big Five Personality traits were measured using the short-form version of the Big Five-Inventory-2 [29]. The questionnaire contains 30 items (6 for each trait), and individuals respond to each statement according to how well it describes them on a scale ranging from 1 (*disagree strongly*) to 5 (*agree strongly*). Mean composites were computed for extraversion (e.g., "I am someone who is outgoing, sociable;" α = .71, *M* = 3.27, *SD* = .72), agreeableness (e.g., "I

am someone who is compassionate, has a soft heart;" α = .81, *M* = 3.85, *SD* = .71), conscientiousness (e.g., "I am someone who is persistent, works until the task is finished;" α = .80, *M* = 3.93, *SD* = .73), negative emotionality (e.g., "I am someone who worries a lot;" α = .84, *M* = 2.37, *SD* = .84), and open-mindedness (e.g., "I am someone who is fascinated by art, music, or literature;" α = .73, *M* = 3.37, *SD* = .73).

Within each of the broader Big Five personality traits are three facets—smaller, more specific descriptors of personality. We also calculated the means for three facets (measured with two items each) of extraversion (sociability (α = .63), assertiveness (α = .65), and energy level (α = .68)), agreeableness (compassion (α = .61), respectfulness (α = .57), and trust (α = .61)), conscientiousness (organization (α = .74), productiveness (α = .59), and responsibility (α = .46)), negative emotionality (anxiety (α = .67), depression (α = .67), and emotional volatility (α = .63)), and open-mindedness (aesthetic sensitivity (α = .53), intellectual curiosity (α = .45), and creative imagination (α = .56)).

Sexual fantasies

Sexual fantasies were assessed with the Sexual Fantasies Questionnaire [30], which asked participants to indicate how often they fantasized about each of 40 themes on a scale ranging from 1 (*never*) to 6 (*daily*). In addition to an overall composite of sexual fantasizing (α = .97, *M* = 1.73, *SD* = .90), the responses can be subsumed into four 10-item composite scales: exploratory sexual fantasies (e.g., "participating in an orgy;" α = .94, *M* = 1.52, *SD* = .94), intimate sexual fantasies (e.g., "making love outdoors in a romantic setting;" α = .93, *M* = 2.45, *SD* = 1.21), impersonal sexual fantasies (e.g., "watching others have sex;" α = .91, *M* = 1.58, *SD* = .90), and sadomasochistic sexual fantasies (e.g., "being forced to do something;" α = .95, *M* = 1.37, *SD* = .89).

Analytical Approach

We began by running bivariate associations between personality traits, facets, and the different sexual fantasy outcomes (i.e., a composite total and each of the four composite scales). Then, to account for the covariation, we ran follow-up linear regressions that included all personality traits, age, and gender. From these models, partial correlations were extracted. For these models that featured control variables, we ran two models, one for the Big Five traits and another for the (15) facets. These were done separately because the broader Big Five are made up of the facets and including them all in one model would introduce multicollinearity problems. This information is presented in Table 1 in the form of partial correlations.

Results

With our sample size of 5,225 people, we could detect effects as small as $r = .039$ with 80% power at $\alpha = .05$. Because of the large sample size, many small correlations (even those slightly smaller than $r = .039$) were statistically significant at $p < .05$ (all $rs > |.03|$ and $rs_p > |.027|$). Thus, we primarily focus on the magnitude of the correlations and highlight the largest and most robust associations.

As seen in Table 1, the most consistent predictor of sexual fantasies was conscientiousness. Higher levels of conscientiousness were associated with a lower frequency of all four types of sexual fantasies (exploration, intimate, impersonal, and sadomasochistic). The next most consistent predictor was agreeableness; higher levels of agreeableness associated with a lower frequency of most types of fantasies. Extraverts tended to fantasize about all four types of sexual fantasies more, but only *after* the covariation between traits was controlled for. People with higher levels of negative emotionality tended to fantasize about all four types of sexual fantasies. Open-mindedness was mostly unrelated to fantasies, particularly after controlling for

covariation with other traits, age, and gender. One consistent pattern that emerged is that associations between personality traits and sexual fantasy were reduced after controlling for covariation between the traits and demographics (i.e., comparing the bivariate and partial correlation columns in Table 1).

Table 1. Bivariate and Partial Correlations between Personality and Sexual Fantasies

Construct	Fantasy Total		Exploration		Intimate		Impersonal		Sadomasochistic	
	Bivariate	Partial	Bivariate	Partial	Bivariate	Partial	Bivariate	Partial	Bivariate	Partial
Extraversion	-0.001	0.058	-0.033	0.037	0.081	0.085	-0.034	0.037	-0.041	0.041
Agreeableness	-0.228	-0.083	-0.243	-0.098	-0.123	-0.015	-0.260	-0.110	-0.233	-0.089
Conscientiousness	-0.265	-0.131	-0.278	-0.141	-0.158	-0.068	-0.270	-0.119	-0.286	-0.150
Negative Emotionality	0.171	0.038	0.173	0.023	0.100	0.051	0.180	0.042	0.189	0.016
Open Mindedness	-0.005	0.023	-0.033	0.008	0.080	0.075	-0.042	0.004	-0.052	-0.026
Sociability	0.004	0.061	-0.007	0.057	0.040	0.051	-0.016	0.053	-0.008	0.058
Assertiveness	0.004	-0.031	-0.020	-0.035	0.073	0.001	-0.011	-0.033	-0.045	-0.047
Energy Level	-0.011	0.086	-0.046	0.064	0.067	0.087	-0.047	0.064	-0.039	0.081
Compassion	-0.201	-0.021	-0.212	-0.024	-0.111	-0.002	-0.226	-0.029	-0.207	-0.026
Respectfulness	-0.258	-0.085	-0.271	-0.093	-0.149	-0.034	-0.276	-0.085	-0.272	-0.103
Trust	-0.115	0.028	-0.126	0.024	-0.052	0.026	-0.148	0.006	-0.108	0.045
Organization	-0.187	0.005	-0.194	0.005	-0.126	-0.008	-0.183	0.016	-0.193	0.010
Productiveness	-0.205	-0.034	-0.221	-0.037	-0.106	-0.017	-0.216	-0.035	-0.230	-0.038
Responsibility	-0.283	-0.055	-0.294	-0.061	-0.168	-0.016	-0.289	-0.054	-0.306	-0.071
Anxiety	0.070	-0.081	0.075	-0.087	0.032	-0.025	0.078	-0.085	0.081	-0.101
Depression	0.384	0.276	0.380	0.270	0.263	0.171	0.382	0.274	0.402	0.290
Emotional Volatility	0.185	0.003	0.182	-0.005	0.123	0.013	0.190	0.007	0.196	-0.007
Aesthetic Sensitivity	0.039	0.055	0.020	0.054	0.086	0.055	0.004	0.043	0.012	0.037
Intellectual Curiosity	-0.005	-0.020	-0.025	-0.021	0.064	0.005	-0.030	-0.025	-0.050	-0.041
Creative Imagination	-0.051	-0.010	-0.078	-0.026	0.037	0.022	-0.078	-0.016	-0.092	-0.027

Note. Correlations $r > |.03|$ and $r_p > |.027|$ are significant at $p < .05$. More intensely green cells are more strongly positive correlations. More intensely red cells are more strongly negative correlations.

In the bottom portion of Table 1 (run in separate models than the higher-order traits), the facet most largely correlated with higher levels of frequent fantasizing in all categories was depression (a facet of negative emotionality), such that people with more depressive personalities tended to fantasize about all four types of sexual fantasies. Respectfulness (for agreeableness) and responsibility (for conscientiousness) had the strongest negative associations with each type of sexual fantasy, although the magnitude of these associations was reduced dramatically in the covariate analysis. Most of the remaining facet associations had correlations smaller than $r = |.20|$, and many associations either became non-significant or even switched signs (albeit these associations were near-zero) after controlling for demographic characteristics and the covariation with the other facets. One surprising finding is that the open-mindedness facets, including creative imagination, were largely unrelated to any type of sexual fantasy, which one might expect because open people tend to fantasize about both sexual and non-sexual things more often [26, 31].

Discussion

We examined associations between sexual fantasies and the Big Five personality traits and facets. People high in agreeableness and conscientiousness reported less frequent sexual fantasies, both overall and for specific types of fantasies (e.g., exploration, intimate, impersonal, sadomasochistic). People high in negative emotionality tended to fantasize more. Measuring facets proved worthwhile in that we were able to discern the parts or facets of these traits that are related to sexual fantasy. Specifically, those with depressive personalities reported more frequent sexual fantasies. Those who were high in respectfulness and responsibility reported less frequent sexual fantasies. One implication of the current work is that individual differences in personality might be useful in predicting variation in sexual fantasy frequencies, although they are not

wholly redundant with each other (and some associations are relatively small or modest). Knowing these associations further advances the predictive power of personality while showing that variation in sexual fantasies are common.

Big Five personality and sexual fantasies

Despite most people experiencing sexual fantasies, sexual fantasies are relatively understudied or framed as pathological [1, 9]. Research shows the utility in approaching the study of sexual fantasies in a value-neutral way has revealed that sexual fantasies are often linked to positive relationship and life outcomes and are also common across the lifespan [7, 12-15]. There are relatively few studies examining associations between psychological predictors, like the Big Five personality traits [20]. However, those studies largely showed that people higher in agreeableness and conscientiousness are less likely to report sexual fantasizing, people higher in openness might be more likely to have sexual fantasies in some contexts, and people high in negative emotionality were more likely to have a variety of sexual fantasies [10, 20, 22, 25, 26].

We were able to replicate some of these findings but not others. Indeed, people higher in agreeableness or conscientiousness tended to report less frequent sexual fantasies. Although negative emotionality was associated with more frequent sexual fantasies (and sexual fantasies of different types), these associations dropped to near-zero (and in many cases non-significance). Openness to experience was largely unrelated to sexual fantasies (except a positive association with intimate sexual fantasies). Extraversion had a small positive association with sexual fantasies, but this mostly only occurred in the models with covariates (and the signs of the associations changed directions). Thus, the main take-aways for the broader trait level are that

agreeableness and conscientiousness are negatively related to sexual fantasy frequencies; extraversion and negative emotionality are positively related to sexual fantasy frequencies—although it mostly depends on whether covariates are controlled for; and openness is mostly unrelated to sexual fantasies. These findings were seen across different types of fantasies, whether they were exploratory, intimate, impersonal, or sadomasochistic in nature. Agreeableness and conscientiousness are associated with norm endorsement, harm prevention, and traditionalism [32, 33]. Thus, it seems reasonable to assume that they might be less likely to engage in fantasies that are non-traditional, bridge social norms, or simulate consensual aggression.

The findings with respect to negative emotionality were a bit harder to interpret. People high in negative emotionality are more likely to focus on negative stimuli, perseverate, and have negative thoughts overall [34, 35]. They are also more likely to experience various types of disgust, including sexual disgust [25, 36]. These observations together might be why they are more likely to think about certain types of sexual fantasies (e.g., sadomasochistic), but this does not explain why they are prone to more ostensibly positive or neutral fantasies as well. However, our study merely examined the frequency of sexual fantasies and not their utility. In other words, some researchers have suggested that people high in negative emotionality might engage in sexual fantasies as an emotion regulation tool to compensate for negative mood [24]. Indeed, when sexual fantasies are operationalized in terms of valence (e.g., positive or negative fantasies, people high in negative emotionality tend to have both positive and negative sexual thoughts and fantasies [24, 25]. Thus, people high in negative emotionality tend to fantasize more overall, such that they have both positive and negative sexual fantasies.

Facets and fantasy

Examining associations between Big Five personality facets (or subcomponents of the broader traits) enabled a more focused examination. Knowing more about these subcomponents has significantly advanced our understanding of personality-outcome associations [28, 37-39]. Although some of the Big Five traits were associated with sexual fantasies, it is possible that there are subcomponents of those larger traits that can give us a clue for why people fantasize more or less.

Indeed, respectfulness (for agreeableness) and responsibility (for conscientiousness) were the drivers of the negative associations seen among their higher-order traits. What this reveals is that agreeable people may be less likely to sexually fantasize because of their respect for norms and others (hence the less common sadomasochistic fantasies) and not that tender feelings about others or perceptions of trust are holding them back from doing so (particularly in the covariate analyses). Likewise, people high in responsibility are likely to moralize themselves and others and avoid sexual thinking (and maybe sex) altogether [18]. What the facet analysis also tells us is that people who are more or less organized (or more or less productive) are no more or less likely to sexually fantasize. The large association with depression (a facet of negative emotionality) can also reveal some reasons why negative emotionality was positively associated with sexual fantasies. Specifically, the fact that depression was strongly related to sexual fantasy—and that anxiety and emotional volatility were not—provides support for the possible emotional regulatory function of sexual fantasies [24, 25]. In other words, it is not the case that emotionally volatile people or anxious people were more likely to sexually fantasize (if anything, anxious people were slightly less likely to fantasize in some models). Rather, those more prone to negative emotionality were also those most likely to sexually fantasize, possibly as a way to have more positive cognitions [25]. The fact that open-mindedness was largely unrelated to sexual

fantasies was a bit surprising, particularly given past research [26, 31]. There is a precedent for null results in this literature [27], and future research can examine if these results might be attributable to the way open-mindedness is operationalized [40].

Limitations and future directions

This study had many strengths, including having a large sample of adults providing information on their personality facets and a variety of sexual fantasies. Nevertheless, some limitations must be discussed.

First, we relied entirely on self-report data, which may be prone to bias [41], particularly when discussing sensitive topics like sexual fantasies, which many people find embarrassing or uncomfortable to discuss [2]. Worth noting, associations between personality and sexual fantasies and thoughts tend to transcend differences in social desirability [24]. Also, the anonymity of our questionnaire likely also reduced these concerns. Nevertheless, future research can investigate ways to circumvent the social desirability effects that accompany self-reports about sexual fantasies. We also hope that this future work recruits a more diverse sample, including those from non-US countries and among people with different relationship statuses (e.g., single people, partnered people, non-monogamous people).

Second, our data were cross-sectional and was merely a snapshot of people’s sexual fantasies and how they were related to personality traits. Stability information on sexual fantasies is relatively rare, but they do tend to fluctuate over the course of a few weeks and months [42, 43] and tend to differ across the lifespan [7]. Future research can follow participants longitudinally to determine if personality traits prospective development of sexual fantasies. Further, it is possible that personality traits and sexual fantasies might co-develop together over

time such that fantasies might become more or less common (or different types of fantasies might become more salient) as people's personalities or life circumstances change.

Conclusion

The current study examined associations between personality traits and sexual fantasies. We found that people high in conscientiousness and agreeableness fantasized less, and people high in neuroticism fantasized more. Among the personality facets, those with higher in depressive, and lower in respectfulness and responsibility facets, were more likely to sexually fantasize. Although sexual fantasies are common among the general population [1, 9], we found that people do not homogeneously report various types of sexual fantasy. Knowing which personality traits are associated with different types of sexual fantasies will likely provide clinicians and mental health professionals with additional information in providing sex-positive therapeutic settings that acknowledge people's sexual thoughts—an effort that is increasingly becoming more common [7, 14].

References

1. Leitenberg, H. and K. Henning, *Sexual fantasy*. Psychological bulletin, 1995. **117**(3): p. 469.
2. Ziegler, A. and T.D. Conley, *The importance and meaning of sexual fantasies in intimate relationships*, in *The psychology of love and hate in intimate relationships*, K. Aumer, Editor. 2016. p. 29-45.
3. Wilson, G.D. and R.J. Lang, *Sex differences in sexual fantasy patterns.* Personality and Individual Differences, 1981. **2**(4): p. 343-346.
4. Hawley, P.H. and W.A. Hensley IV, *Social dominance and forceful submission fantasies: Feminine pathology or power?* Journal of Sex Research, 2009. **46**(6): p. 568-585.
5. Leistner, C.E., et al., *The Co-existence of Love and Desire: Experiences of Bisexual, Lesbian, and Heterosexual Women.* Sexuality & Culture, 2022. **26**(2): p. 531-547.
6. Bogaert, A.F., B.A. Visser, and J.A. Pozzebon, *Gender differences in object of desire self-consciousness sexual fantasies.* Archives of Sexual Behavior, 2015. **44**: p. 2299-2310.
7. Kolodziejczak, K., et al., *Sexual activity, sexual thoughts, and intimacy among older adults: Links with physical health and psychosocial resources for successful aging.* Psychology and aging, 2019. **34**(3): p. 389.
8. Bondü, R. and J.B. Birke, *Aggression-related sexual fantasies: Prevalence rates, sex differences, and links with personality, attitudes, and behavior*. The journal of sexual medicine, 2021. **18**(8): p. 1383-1397.
9. Lehmiller, J.J. and A.M. Gormezano, *Sexual fantasy research: A contemporary review*. Current Opinion in Psychology, 2023. **49**: p. 101496.

10. Lehmiller, J.J., *Tell me what you want: The science of sexual desire and how it can help you improve your sex life.* 2018, Boston: Da Capo Lifelong Books.

11. Muise, A., U. Schimmack, and E.A. Impett, *Sexual frequency predicts greater well-being, but more is not always better.* Social Psychological and Personality Science, 2015. **7**(4): p. 295-302.

12. Busch, T.M., et al., *Exploring the Impact of Fantasizing on Romantic Relationships and Attachment.* Sexuality & Culture, 2024.

13. Birnbaum, G.E., et al., *What Fantasies Can Do to Your Relationship: The Effects of Sexual Fantasies on Couple Interactions.* Personality and Social Psychology Bulletin, 2019. **45**(3): p. 461-476.

14. Newbury, R., et al., *Sexual fantasy as a clinical intervention.* Sexual and Relationship Therapy, 2012. **27**(4): p. 358-371.

15. Kolodziejczak, K., et al., *Perceived importance and enjoyment of sexuality in late midlife: Cohort differences in the Longitudinal Aging Study Amsterdam (LASA).* Sexuality Research and Social Policy, 2021. **18**: p. 621-635.

16. John, O.P. and S. Srivastava, *The Big Five trait taxonomy: History, measurement, and theoretical perspectives*, in *Handbook of personality: Theory and research*, L.A. Pervin and O.P. John, Editors. 1999. p. 102-138.

17. Soto, C.J. and O.P. John, *The next Big Five Inventory (BFI-2): Developing and assessing a hierarchical model with 15 facets to enhance bandwidth, fidelity, and predictive power*. Journal of Personality and Social Psychology, 2017. **113**(1): p. 117-143.

18. Allen, M.S. and E.E. Walter, *Linking big five personality traits to sexuality and sexual health: A meta-analytic review.* Psychological Bulletin, 2018. **144**(10): p. 1081-1110.

19. Bainbridge, T.F., S.G. Ludeke, and L.D. Smillie, *Evaluating the Big Five as an organizing framework for commonly used psychological trait scales*. Journal of Personality and Social Psychology, 2022. **122**(4): p. 749-777.

20. Nowak, B., *Big Five Personality Traits: Sexual Fantasies*, in *Encyclopedia of Sexual Psychology and Behavior*, T.K. Shackelford, Editor. 2023, Springer. p. 1-3.

21. Bivona, J.M., J.W. Critelli, and M.J. Clark, *Women's Rape Fantasies: An Empirical Evaluation of the Major Explanations.* Archives of Sexual Behavior, 2012. **41**(5): p. 1107-1119.

22. Lehmiller, J.J., *Fantasies About Consensual Nonmonogamy Among Persons in Monogamous Romantic Relationships.* Archives of Sexual Behavior, 2020. **49**(8): p. 2799-2812.

23. Sierra, J., S. Álvarez-Castro, and E. Miró, *Relación entre rasgos de personalidad y fantasías sexuales.* Terapia Psicológica, 1995. **3**: p. 7-12.

24. Egan, V. and V. Campbell, *Sensational interests, sustaining fantasies and personality predict physical aggression.* Personality and Individual Differences, 2009. **47**(5): p. 464-469.

25. Moyano, N. and J.C. Sierra, *Relationships between personality traits and positive/negative sexual cognitions.* International Journal of Clinical and Health Psychology, 2013. **13**(3): p. 189-196.

26. Pérez-González, S., N. Moyano, and J.C. Sierra, *La capacidad de ensoñación sexual: su relación con la actitud hacia las fantasías sexuales y rasgos de personalidad.* Cuadernos de Medicina Psicosomática y Psiquiatría de Enlace, 2011. **99**: p. 9-20.

27. Williams, K.M., et al., *Inferring Sexually Deviant Behavior From Corresponding*

Fantasies:The Role of Personality and Pornography Consumption. Criminal Justice and Behavior, 2009. **36**(2): p. 198-222.

28. Schwaba, T., et al., *A facet atlas: Visualizing networks that describe the blends, cores, and peripheries of personality structure*. PLOS ONE, 2020. **15**(7): p. e0236893.

29. Soto, C.J. and O.P. John, *Short and extra-short forms of the Big Five Inventory–2: The BFI-2-S and BFI-2-XS.* Journal of Research in Personality, 2017. **68**: p. 69-81.

30. Wilson, G.D., *The sex fantasy questionnaire: An update.* Sexual and Relationship Therapy, 2010. **25**(1): p. 68-72.

31. Sánchez-Bernardos, M.L., et al., *Fantasy Proneness and Personality Profiles.* Imagination, Cognition and Personality, 2015. **34**(4): p. 327-339.

32. Wilmot, M.P. and D.S. Ones, *Agreeableness and its consequences: A quantitative review of meta-analytic findings.* Personality and Social Psychology Review, 2022. **26**(3): p. 242-280.

33. Roberts, B.W., et al., *The structure of conscientiousness: An empirical investigation based on seven major personality questionnaires.* Personnel Psychology, 2005. **58**(1): p. 103-139.

34. Robinson, M.D., et al., *Stuck in a rut: perseverative response tendencies and the neuroticism-distress relationship.* Journal of Experimental Psychology: General, 2006. **135**(1): p. 78.

35. Clark, L.A. and D. Watson, *Temperament: An ongoing paradigm for trait psychology*, in *Handbook of Personality: Theory and Research*, O.P. John, R.W. Robins, and L.A. Pervin, Editors. 2008, The Guilford Press: New York. p. 265-286.

36. Olatunji, B.O., et al., *Core, animal reminder, and contamination disgust: Three kinds of*

disgust with distinct personality, behavioral, physiological, and clinical correlates. Journal of Research in Personality, 2008. **42**(5): p. 1243-1259.

37. Weidmann, R., et al., *Trait and facet personality similarity and relationship and life satisfaction in romantic couples.* Journal of Research in Personality, 2023. **104**: p. 104378.

38. Mund, M. and F.J. Neyer, *Treating personality-relationship transactions with respect: Narrow facets, advanced models, and extended time frames.* Journal of Personality and Social Psychology, 2014. **107**(2): p. 352-368.

39. Stewart, R.D., et al., *The finer details? The predictability of life outcomes from Big Five domains, facets, and nuances.* Journal of Personality, 2022. **90**(2): p. 167-182.

40. DeYoung, C.G., R.G. Grazioplene, and J.B. Peterson, *From madness to genius: The Openness/Intellect trait domain as a paradoxical simplex.* Journal of Research in Personality, 2012. **46**(1): p. 63-78.

41. Paulhus, D.L. and S. Vazire, *The self-report method*, in *Handbook of Research Methods in Personality Psychology*, R.W. Robins, R.C. Fraley, and R.F. Krueger, Editors. 2007, The Guilford Press: New York. p. 224-239.

42. Cascalheira, C.J., et al., *Changes in Sexual Fantasy and Solitary Sexual Practice During Social Lockdown Among Young Adults in the UK.* Sexual Medicine, 2021. **9**(3): p. 100342-100342.

43. Dawson, S.J., K.D. Suschinsky, and M.L. Lalumière, *Sexual Fantasies and Viewing Times Across the Menstrual Cycle: A Diary Study.* Archives of Sexual Behavior, 2012. **41**(1): p. 173-183.

Response to Reviewers

We would like to thank the editor and reviewers for their thoughtful comments on the manuscript. We very much appreciate the constructive feedback and believe that the manuscript has improved significantly as a result of their suggestions. Below, we report how each of the reviewer issues was addressed and the corresponding changes to the manuscript. The reviewer comments are non-bolded, and our responses are bolded. We are happy to make any additional changes recommended by the Editor.

Editor

One reviewer supports the manuscript as is, while the other suggests several minor but important revisions that need to be addressed. These include clarifying the theoretical framework, elaborating on demographic variables, and improving the reporting of limitations. Please revise the manuscript accordingly.

Thank you for handling our manuscript. In evaluating the reviewers' comments, we elected to add details related to the inclusion/exclusion criteria in the Method (p 7) and to add a brief remark on the Implications of the study (see pg 12-13).

Reviewer #1

The paper addresses an important topic related to stress and resilience. However, some areas need improvement. Below is some feedback for improvement.

- Add literature review for second objective association between nurse's demographic characteristics and both stress and resilience (eg: gender, age)
- State what is the theory being used in this study.
- Methodology- What is the inclusion and exclusion criteria for the targeted sample.
- Add more demographic characteristics in the table related to the study (eg: age)
- Limitation, hanging sentence. "As such"
- Add implication and conclusion related to the second objective of the study

We thank the reviewer for taking the time to provide a review. In discussions with the Editor, they partially left it up to our discretion for how to integrate comments from this review. After reviewing the comments, we ultimately decided to add information related to our inclusion/exclusion criteria (see p 7) and about the implications of our study (see pages 12-13). The other remarks fell a bit beyond the purview of our study or did not correspond to our manuscript, so we elected not to make those particular changes.

Reviewer #2

This interesting study of 5,225 adults examined the links between Big Five personality traits and sexual fantasies. High conscientiousness and agreeableness, driven by the facets of respectfulness and responsibility, were associated with fewer fantasies across various domains. Neuroticism, particularly the depression facet, correlated with more frequent fantasizing, while extraversion and open-mindedness showed minimal impact. The well-conducted study's findings reveal how personality shapes sexual thoughts, aiding clinicians in fostering sex-positive therapy. It highlights the diversity of sexual fantasies and supports inclusive approaches to sexual well-being.

We thank the reviewer for their positive evaluation of our manuscript!

Data Review URL

Click here to download Data Review URL

https://osf.io/c9pzj/?view_only=b9672f35521f483c90a84701dfb105fc
