# Supplementary material for: Associations between big five personality traits, facets, and sexual fantasies
Source: PLoS One. 2026 Feb 4;21(2):e0329745. doi: 10.1371/journal.pone.0329745 (PMC12871982; doi:10.1371/journal.pone.0329745)
Supplement: Attachment — Submitted filename: Response061325.docx [file pone.0329745.s003.docx]

**Response to Reviewers**

We would like to thank the editor and reviewers for their thoughtful comments on the manuscript. We very much appreciate the constructive feedback and believe that the manuscript has improved significantly as a result of their suggestions. Below, we report how each of the reviewer issues was addressed and the corresponding changes to the manuscript. The reviewer comments are non-bolded, and our responses are bolded. We are happy to make any additional changes recommended by the Editor.

**Editor**

One reviewer supports the manuscript as is, while the other suggests several minor but important revisions that need to be addressed. These include clarifying the theoretical framework, elaborating on demographic variables, and improving the reporting of limitations. Please revise the manuscript accordingly.

**Thank you for handling our manuscript. In evaluating the reviewers’ comments, we elected to add details related to the inclusion/exclusion criteria in the Method (p 7) and to add a brief remark on the Implications of the study (see pg 12-13).**

**Reviewer #1**

The paper addresses an important topic related to stress and resilience. However, some areas need improvement. Below is some feedback for improvement.
- Add literature review for second objective association between nurse’s demographic characteristics and both stress and resilience (eg: gender, age)
- State what is the theory being used in this study.
- Methodology- What is the inclusion and exclusion criteria for the targeted sample.
- Add more demographic characteristics in the table related to the study (eg: age)
- Limitation, hanging sentence. “As such”
- Add implication and conclusion related to the second objective of the study

**We thank the reviewer for taking the time to provide a review. In discussions with the Editor, they partially left it up to our discretion for how to integrate comments from this review. After reviewing the comments, we ultimately decided to add information related to our inclusion/exclusion criteria (see p 7) and about the implications of our study (see pages 12-13). The other remarks fell a bit beyond the purview of our study or did not correspond to our manuscript, so we elected not to make those particular changes.**

**Reviewer #2**

This interesting study of 5,225 adults examined the links between Big Five personality traits and sexual fantasies. High conscientiousness and agreeableness, driven by the facets of respectfulness and responsibility, were associated with fewer fantasies across various domains. Neuroticism, particularly the depression facet, correlated with more frequent fantasizing, while extraversion and open-mindedness showed minimal impact. The well-conducted study’s findings reveal how personality shapes sexual thoughts, aiding clinicians in fostering sex-positive therapy. It highlights the diversity of sexual fantasies and supports inclusive approaches to sexual well-being.

**We thank the reviewer for their positive evaluation of our manuscript!**
