# Supplementary material for: Associations between big five personality traits, facets, and sexual fantasies
Source: PLoS One. 2026 Feb 4;21(2):e0329745. doi: 10.1371/journal.pone.0329745 (PMC12871982; doi:10.1371/journal.pone.0329745)
Supplement: Attachment — Submitted filename: Response111825.docx [file pone.0329745.s005.docx]

**Response to Reviewers**

We would like to thank the editor and reviewers for their thoughtful comments on the manuscript. We very much appreciate the constructive feedback and believe that the manuscript has improved significantly as a result of their suggestions. Below, we report how each of the reviewer issues was addressed and the corresponding changes to the manuscript. The reviewer comments are non-bolded, and our responses are bolded. We are happy to make any additional changes recommended by the Editor.

**Editor**

Both Reviewers and I think that the manuscript can be accepted for publication. However, as suggested by Reviewer #3, they are invited to (1) check whether all the references used are appropriately related to the topic of this article; (2) provide a more detailed explanation of the figure to enhance clarity and interpretability; (3) to strengthen the concluding statements to summarize the study’s findings succinctly and clearly articulate the novelty of the work.

Thus, I invite the authors to resubmit their revised manuscript as soon as possible, in line with the points outlined above, for acceptance.

**Thank you for serving as editor for this paper, expediting its review, and for the positive evaluation! We have now removed references that were tangentially related to the point being made near it in the text, inserted a stand-alone paragraph describing the table/figure, and revised the conclusion to more succinctly state the study findings while expanding our discussion on the novelty of this work and its importance for motivating more constructive conversations across domains. These changes are all detailed below in response to each reviewer comment.**

**Reviewer #3**

1.) I have carefully examined the improvements made by the researchers to conclude that this article can be accepted and published by the PLOS ONE Journal.

However, researchers must make improvements to relate the references used better to the topic of this article.

**Thank you for your positive evaluation of our manuscript. We now see that it might some of the citations might have too-distant a connection to what they are supporting in the text. We have now gone through and either added additional textual information (to more closely link the reference with the text) or removed/replaced a particular citation. These small changes can be seen throughout the manuscript, particularly in the Introduction and Discussion (see marked version of the manuscript).**

2.) The figure should be explained in more detail to make it easy to understand.

**We have now added a stand-alone paragraph describing Table 1 (see p 9, lns 184-189). We also expanded the table/figure note to be clearer about what the colors corresponded to. Here is that paragraph reproduced:**

**“Table 1 presents a heat map of the associations between Big Five personality traits and sexual fantasy frequencies (both bivariate and after partialling out the other traits, age, and gender). More intensely green colors correspond to more positive correlations. More intensely red colors correspond to more negative correlations. Varying shades of yellow correspond to values between these intensely positive and negative correlations and are typically smaller and closer to zero. Bivariate and partial correlations greater than |.03| were significant at *p* = .05.”**

3.) The conclusions compiled in paragraphs may need to be improved to explain the results of this study briefly and affirm the existence of novelty in this article.

*** I found the strengths of this article, but there are still weaknesses that need to be fixed.

This article is Acceptable in the PLOS ONE Journal.

**We have now more succinctly summarized the results of our study and more squarely focused the contribution of our work. We then make a linkage between this knowledge gained with how it can be leveraged to promote informed conversation in a variety of settings. This paragraph can be found on lns 322-335:**

**“The present study demonstrated that personality meaningfully relates to patterns of sexual fantasy. At the broad trait level, conscientiousness and agreeableness were associated with lower fantasy frequency, whereas neuroticism predicted greater fantasy engagement. Importantly, examining personality facets revealed more specific psychological correlates of fantasy, with higher depressive tendencies and lower responsibility and respectfulness emerging as the most consistent predictors. This facet-level approach offers a more nuanced understanding of who fantasizes and how. This extends past work that has relied primarily on global traits and provides a preliminary assessment of how sexual fantasies and related to novel and more specific characteristics. Because sexual fantasies are common yet highly variable across individuals, identifying personality correlates may help clinicians and educators support more informed, sex-positive conversations that acknowledge differences in sexual thought and expression. Future work should continue refining these associations and examine whether personality dynamics predict changes in fantasy over time or across relational contexts.”**

**Thank you again for your positive assessment of our manuscript.**

**Reviewer #4**

1.) The study presents the results of original research. Results reported have not been published elsewhere. Experiments, statistics, and other analyses are performed to a high technical standard and are described in sufficient detail. Conclusions are presented in an appropriate fashion and are supported by the data. The article is presented in an intelligible fashion and is written in standard English. The research meets all applicable standards for the ethics of experimentation and research integrity. The article adheres to appropriate reporting guidelines and community standards for data availability.

**Thank you for your thorough review of our manuscript. We appreciate your recognition of how we contextualized the results and of our writing. We also feel that our revised concluding paragraph provides a succinct take-away of our findings and the important implications for having critical policy discussions. This paragraph can be found on lns 322-335:**

**“The present study demonstrated that personality meaningfully relates to patterns of sexual fantasy. At the broad trait level, conscientiousness and agreeableness were associated with lower fantasy frequency, whereas neuroticism predicted greater fantasy engagement. Importantly, examining personality facets revealed more specific psychological correlates of fantasy, with higher depressive tendencies and lower responsibility and respectfulness emerging as the most consistent predictors. This facet-level approach offers a more nuanced understanding of who fantasizes and how. This extends past work that has relied primarily on global traits and provides a preliminary assessment of how sexual fantasies and related to novel and more specific characteristics. Because sexual fantasies are common yet highly variable across individuals, identifying personality correlates may help clinicians and educators support more informed, sex-positive conversations that acknowledge differences in sexual thought and expression. Future work should continue refining these associations and examine whether personality dynamics predict changes in fantasy over time or across relational contexts.”**

**Thank you again for your positive assessment of our manuscript.**
